# PrMFTP: Multi-functional therapeutic peptides prediction based on multi-head self-attention mechanism and class weight optimization

**DOI:** 10.1371/journal.pcbi.1010511

**Published:** 2022-09-12

**Authors:** Wenhui Yan, Wending Tang, Lihua Wang, Yannan Bin, Junfeng Xia

**Affiliations:** Information Materials and Intelligent Sensing Laboratory of Anhui Province and Key Laboratory of Intelligent Computing and Signal Processing of Ministry of Education, Institutes of Physical Science and Information Technology, Anhui University, Hefei, Anhui, China; Universita degli Studi di Torino, ITALY

## Abstract

Prediction of therapeutic peptide is a significant step for the discovery of promising therapeutic drugs. Most of the existing studies have focused on the mono-functional therapeutic peptide prediction. However, the number of multi-functional therapeutic peptides (MFTP) is growing rapidly, which requires new computational schemes to be proposed to facilitate MFTP discovery. In this study, based on multi-head self-attention mechanism and class weight optimization algorithm, we propose a novel model called PrMFTP for MFTP prediction. PrMFTP exploits multi-scale convolutional neural network, bi-directional long short-term memory, and multi-head self-attention mechanisms to fully extract and learn informative features of peptide sequence to predict MFTP. In addition, we design a class weight optimization scheme to address the problem of label imbalanced data. Comprehensive evaluation demonstrate that PrMFTP is superior to other state-of-the-art computational methods for predicting MFTP. We provide a user-friendly web server of PrMFTP, which is available at http://bioinfo.ahu.edu.cn/PrMFTP.

## Author summary

Therapeutic peptides possess a wide range of biological properties, including anti-cancer, anti-hypertensive, anti-viral, and so forth. This is a prerequisite to understanding functional therapeutic peptides and ultimately designing these peptides for drug discovery and development. With the number of multi-functional therapeutic peptides (MFTP) growing, predicting these peptides is an urgent problem in the development of novel peptide-based therapeutics. We develope PrMFTP, an approach for MFTP prediction based on multi-label classification. Our method uses a deep neural network and multi-head self-attention that are able to optimize the features from the peptide sequences. Furthermore, for the imbalance problem in the multi-label dataset, a novel class weight optimization scheme is used to improve the performance of PrMFTP. We evaluate our approach using example-based measures and compare it with the top-performing MLBP method as well as the SOTA multi-functional peptides prediction approaches, demonstrating the improvement of PrMFTP over the existing methods.

## 1. Introduction

Over the last decades, the number of peptide drug approvals has increased steadily, and the global peptide therapy market has an average growth rate of 7.7% [1]. Peptide drugs have been used to treat cancer, diabetes, HIV infection and so on [1, 2]. Compared with proteins and antibodies, therapeutic peptides have many advantages as potential therapeutic drugs: low production cost, low toxicity, and room temperature storage [3]. With the development of sequencing technologies and peptide synthesis methods in the post-genome era, more and more therapeutic peptides, with two or more functional characteristics, have been found. These multi-functional therapeutic peptides (MFTP) are very important for new peptide drug design. However, traditional experimental methods to screen therapeutic peptides are expensive and time-consuming, a fast and effective computational approach could be an excellent alternative [4].

Data-driven computational methods, especially machine learning (ML) methods, have been widely used in the prediction of therapeutic peptides [5–10]. Thus far, random forest, extra trees and extreme gradient boosting algorithms have successfully identified tumor homing peptide (THP) [11], anti-cancer peptide (ACP) [12], and anti-parasitic peptide (APP) [13]. Furthermore, TPpred-ATMV used BioSeq-BLM tool to extract various peptide sequence features to optimize the prediction of therapeutic peptides [14, 15]. Among these traditional ML methods, suitable feature sets are very important to distinguish functional and nonfunctional peptides and achieve excellent performance. However, manual feature selection requires prior knowledge, and high-dimensional features may cause overfitting. Recently, with the development of artificial intelligence technology, the importance and advantage of deep learning (DL) methods in the field of bioinformatics have been well demonstrated [16–21]. Various DL methods have been utilized for therapeutic peptides prediction [22–25], such as Fang *et al*. proposed a predictor based on DL combined with a character embedding layer for anti-fungal peptides identification [24], Li *et al*. developed a dual-channel deep neural network (DNN) model for identifying variable-length antiviral peptides [25]. Compared with the traditional ML methods that need to extract or select features manually, the DL models can automatically learn the feature representation with limited peptide knowledge [26]. Overall, numerous methods based on ML or DL have been proposed to predict the therapeutic peptides, but most of them focused on the mono-functional peptides, and could not rapidly and efficiently identify the therapeutic peptides with two or more functional characteristics. A multi-label classification model of therapeutic peptides prediction may make up for the shortcomings.

To date, many multi-label classification algorithms have been proposed [4], and they can be divided into two categories: problem transformation and algorithm adaptation. (1) Problem transformation approach transforms the multi-label learning problem into a more single-label classification [27]. For example, multi-label classification can be transformed into multiple binary classifications by binary relevance (BR) [28], or label ranking tasks by calibrated label ranking (CLR) [29]. Furthermore, the random k-labelsets method (RKL) regards each independent label subset in the multi-label dataset as a new label, and classifies the datasets with the new labels [30]. Among these three algorithms, BR is the easiest one, but ignores the correlation between labels. CLR only considers the correlation between two labels. RKL considers the correlations among labels. However, RKL turns the multi-label problem into multi-classification, which is easy to cause labels in the test set not to appear in the training set. In addition, it may increase the model complexity. (2) Algorithm adaptation approach solves the multi-label learning problem by directly processing multi-label data using popular learning technologies [27], such as we previously proposed a DL-based multi-label approach for determining the multi-functional bioactive peptides [4], and Wu *et al*. proposed robust low-rank learning of jointing ranking support vector machine and binary relevance (RBRL) for text, images, music and bioinformatics fields [31].

In this study, for MFTP identification, we proposed PrMFTP, a novel multi-label predictor based on DNN and multi-head self-attention mechanism (MHSA) [32]. PrMFTP model used MHSA to optimize and filter the features extracted from the deep network layer, so as to improve the prediction performance. For the class imbalance problem in the multi-label dataset, we proposed a novel class weight optimization method to learn complex characteristics from data. Compared with resampling methods, our method changed the loss value by adding new class weights for different classes to deal with the imbalance in the data set.

## 2. Materials and methods

### 2.1 Datasets

In order to train and evaluate our proposed model, we constructed a benchmark MFTP dataset. As July 2021, we conducted a literature query on Google Scholar with the keyword ‘therapeutic peptide’ and obtained 22 kinds of therapeutic peptide sequence datasets. There are a total of 22 types of therapeutic peptides: AAP [33], anti-bacterial peptide (ABP) [33, 34], ACP [12, 34], anti-coronavirus peptide (ACVP) [9], anti-diabetic peptide (ADP) [35], anti-endotoxin peptide (AEP) [34], anti-fungal peptide (AFP) [24, 34], anti-HIV peptide (AHIVP) [34], anti-hypertensive peptide (AHP) [5], anti-inflammatory peptide (AIP) [33, 36], anti-MRSA peptide (AMRSAP) [34], anti-parasitic peptide (APP) [13, 34], anti-tubercular peptide (ATP) [37], anti-viral peptide (AVP) [33, 34], blood-brain barrier peptide (BBP) [7], biofilm-inhibitory peptide (BIP) [8, 34], chemotactic peptide (CP) [34], cell-penetrating peptide (CPP) [33], dipeptidyl peptidase IV peptide (DPPIP) [38], quorum-sensing peptide (QSP) [33], surface-binding peptide (SBP) [33] and THP [11].

These datasets were processed according to the three criteria: (1) the peptides with sequence contained non-standard amino acids were abandoned; (2) the peptides with sequence length less than 5bp or more than 50bp were deleted. The reason is that long peptides are generally toxic and have low stability, while very short peptide sequences do not have good activity [39]; (3) the peptides with their number less than 40 were removed. In addition, CP was abandoned since there are too few CP to be statistically significant [34]. After these processes, we combined theses therapeutic peptide data and assigned the peptides with multi-label functions. A benchmark dataset was obtained, of which 8,415 peptides belong to one functional attribute, 981 with two different functional attributes, 329 with three different functional attributes, 91 with four different functional attributes, 31 with five different functional attributes and 27 with more than five different functional attributes. The summary of different therapeutic peptide data is shown in Table 1, and the details of the multi-label dataset are summarized in S1 Fig. We sampled the training set with a ratio of 80% in this dataset, whereas the remaining 20% data were applied as the test set.

**Table 1:** The summary of the dataset used in this work.

Type	Original number	Final number	Type	Original number	Final number
AAP	135	133	APP	319	279
ABP	2,469	2,154	ATP	246	242
ACP	1,052	1,043	AVP	736	711
ACVP	137	126	BBP	119	117
ADP	509	509	BIP	339	333
AEP	70	58	CPP	462	459
AFP	2,324	1,352	DPPIP	313	313
AHIVP	109	101	QSP	220	220
AHP	948	917	SBP	104	104
AIP	2,049	2,049	THP	651	651
AMRSAP	173	168	**Total**	**11,142**	**9,841**

### 2.2 PrMFTP framework

**Fig 1:**
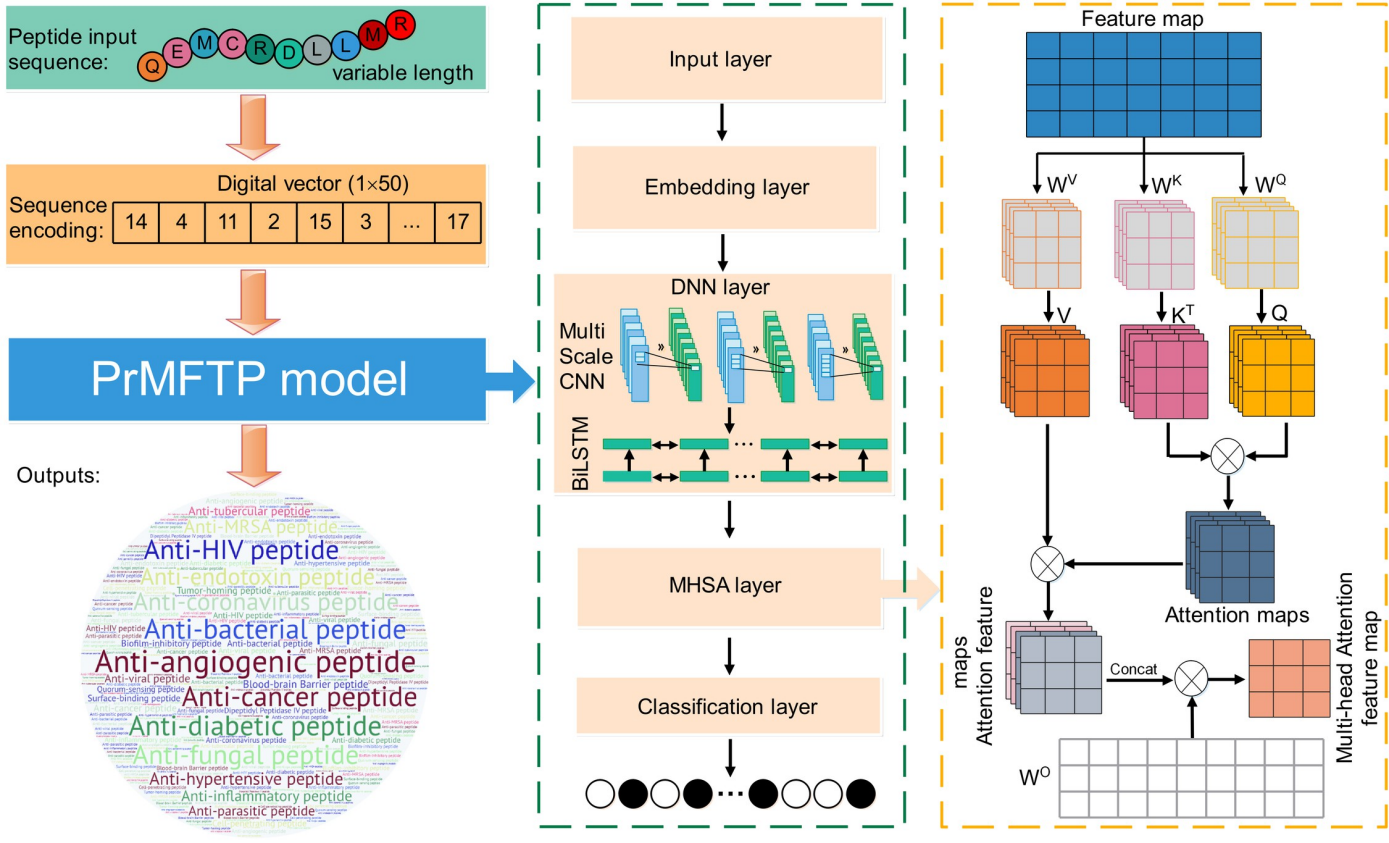
The framework of PrMFTP. First, peptide sequences are encoded as an input vector using numbers, and converted into a fixed-size matrix through the embedding layer. Second, DNN layer, a combination of multi-scale CNN and BiLSTM architectures, is used to capture the sequence features. Third, multi-head self-attention mechanism (MSHA) is used to make the model attend the more important and discriminating sequence features for prediction of multi-functional therapeutic peptides. Finally, the resulting feature matrix is fed into a classification layer and applied to score the different therapeutic peptides to achieve the predicted result.

The framework of PrMFTP is shown in Fig 1, which consists of five layers: input layer, embedding layer, DNN layer, MHSA layer, and classification layer. The details of these layers are described as follows:

### Input layer

This layer encoded peptide sequence into a digital vector. The peptide sequences consisted of 20 standard amino acids {A, C, D, E, F, G, H, I, M, N, P, Q, R, S, T, V, W, Y}, and these amino acids were encoded into nature numbers {1, 2, 3, 4, 5, 6, 7, 8, 9, 10, 11, 12, 13, 14, 15, 16, 17, 18, 19, 20}, respectively. In our benchmark dataset, the length of the peptide sequences varied from 5 bp (the minimum length) to 50 bp (the maximum length), but the model could only process peptide sequences with a fixed dimension. Therefore, if the length of a peptide sequence is less than 50 bp, the pad ones were set to 0.

### Embedding layer

Through the embedding layer, the sequence vector obtained from the input layer was transformed into a dense continuous feature vector. The embedding layer algorithm encapsulated as much information in the peptide sequence text as possible into the vector space [40]. Finally, the peptide sequence was represented by the embedding matrix, which was used as the input matrix to DNN layer.

### DNN layer

The DNN layer consisted of a multi-scale convolutional neural network (CNN) and bi-directional long short-term memory (BiLSTM). Firstly, the multi-scale convolutional layer was used to extract the semantic features of the sequence. To obtain more comprehensive features, the convolution windows with sizes 2, 3 and 8 were used to extract the peptide features of different sequence lengths. Then, with the convolution feature matrix, the maximum pooling operation was used to reduce the number of features and prevent over-fitting. Secondly, feature matrix extracted from CNN was used as the input matrix to BiLSTM. We used BiLSTM to extract the hidden information in sequences, and it can also achieve long-dependent sequence information. The core of BiLSTM was to use memory cells to remember long-term historical information that could be impressed with memory cells and managed with a door mechanism. The door structure was used to limit the amount of information. BiLSTM effectively captured the relationship between the properties of the sequence in the forward and backward directions to obtain global information from the sequences [41]. Finally, sequence feature matrix extracted from BiLSTM was used as the input matrix to MHSA layer.

### MHSA layer

MHSA was proposed to focus attention on different parts of the peptide sequence. Therefore, in our model architecture, MHSA layer further optimized the sequence features filtered by DNN layer to capture evolutionary features. MHSA was composed of multiple self-attention (SA) mechanisms, which were used to represent the context of learning sequences. The mathematical description of SA is as follows:

$$\left\{ \begin{array}{c} Q=F{*W}^{Q}\\ K=F*W^{K}\\ V=F{*W}^{V} \end{array}\right.$$ (1)

$$Attention\;(Q,K,V)=softmax\left(\frac{Q*K^{T}}{\sqrt{d_{k}}}\right)V$$ (2)

where $F\in R^{L\times d_{m}}$ is the output matrix of DNN layer, and $Q,K,V\in R^{L\times d_{k}}$ are the query, key and value matrix, respectively. These matrixes are obtained by *F* through a linear transformation with $W^{Q},W^{K},W^{V}\in R^{d_{m}\times d_{k}}$, here *d*_*m*_ is twice the dimensionality of the BiLSTM hidden layer, *d*_*k*_ is the dimension of the query, key or value vector and *L* is the length of an input sequence.

Based on the SA mechanism, the linear matrix was changed from one set (*W*^*Q*^, *W*^*K*^, *W*^*V*^) to multiple sets {$\left(W_{0}^{Q},W_{0}^{K},W_{0}^{V}\right)$, …, $\left(W_{i}^{Q},W_{i}^{K},W_{i}^{V}\right)$}. Different randomly initialized linear matrices (*W*^*Q*^, *W*^*K*^, *W*^*V*^) can map input vectors to different subspaces, allowing the model to understand input information from different spatial dimensions. Therefore, the mathematical description of MHSA is as follows:

$${Head}_{i}=Attention(QW_{i}^{Q},KW_{i}^{K},VW_{i}^{V}),i=1,\cdots ,h$$ (3)

$$MHSA(Q,K,V)=W^{O}*concat({head}_{1},\cdots ,{head}_{h})$$ (4)

where $W_{i}^{Q},W_{i}^{K},W_{i}^{V}\in R^{d_{m}\times d_{k}}$ are the query, key and value matrixes of the *i*-th head, respectively, *h* is the size of heads and $W^{O}\in R^{d_{m}\times hd_{k}}$ is a linear transformation matrix to map the output of MHSA to the space of the same dimension.

### Classification layer

We used the full connection layer as the classification layer. The vector from the fully connected layer was used as the input of the output layer. In the multi-label problem, the probability of each node was independent with each other, and binary cross-entropy was used as the loss function. Taking sigmoid as the activation function, the score of each node between 0 and 1 was obtained. Finally, we used the threshold of 0.5 to get the prediction label of each category.

### 2.3 Class weights

In this work, the multi-label dataset is imbalanced, in which some therapeutic peptides are very frequent (for example, the number of therapeutic peptide in the largest class (ABP) is 2,154), while others are quite rare (for example, the number in the smallest one (AEP) is 58). Therefore, we proposed a class weight optimization method to add class weights to different labels, with the prupose of overcoming the imbalanced problem. We named this novel calculation method as CW, and its mathematical description is as follows:

$$CW:\;W_{i}=\varphi *{\left[\log\frac{N}{n_{i}}\right]}^{\theta }$$ (5)

where *W*_*i*_ is the weight of the *i*-th class, *N* is the total number of instances in the training set, *n*_*i*_ is the number of instances that are associated with the *i*-th class, *φ* is a hyperparameter whose purpose is to increase the loss value of the model by doubling the label weight of each category, and θ is a constant, and its constraints are as shown in Formula (6):

$$1\leq \theta \leq \frac{\ln\;X-\ln\;Y}{\ln\;\log_{Y}\;X}$$ (6)

where *X* is obtained by dividing *N* by the minimum value of *n*_*i*_, and *Y* is obtained by *N* by maximum value of *n*_*i*_.

### 2.4 Performance metrics

As illustrated in the previous works on multi-label classifications [42–44], several evaluation indexes have been proposed to evaluate the model performance. In this work, the performance of our proposed multi-label models is estimated by Precision, Coverage, Accuracy, Absolute true, and Absolute false. The mathematical description of these measurements is as follows:

$$\left\{ \begin{array}{c} \mathrm{Precision}=\frac{1}{N}\sum_{i=1}^{N}\frac{\left\|L_{i}⋂L_{i}^{*}\right\|}{\left\|L_{i}^{*}\right\|}\\ \mathrm{Coverage}=\frac{1}{N}\sum_{i=1}^{N}\frac{\left\|L_{i}⋂L_{i}^{*}\right\|}{\left\|L_{i}\right\|}\\ \mathrm{Accuracy}=\frac{1}{N}\sum_{i=1}^{N}\frac{\left\|L_{i}⋂L_{i}^{*}\right\|}{\left\|L_{i}⋃L_{i}^{*}\right\|}\\ \mathrm{Absolute}\;\mathrm{true}=\frac{1}{N}\sum_{i=1}^{N}\Delta (L_{i},L_{i}^{*}\|\\ \mathrm{Absolute}\;\mathrm{false}=\sum_{i=1}^{N}\frac{\left\|L_{i}⋃L_{i}^{*}\|-\|L_{i}⋂L_{i}^{*}\right\|}{M} \end{array}\right.$$

where *N* is the total number of multi-functional therapeutic peptide sequences in the datasets, *M* represents the number of labels, that is the function types of therapeutic peptides ∩/∪ denotes the intersect/union in the set theory, ‖∙‖ indicates the operation of calculating the number of elements, *L*_*i*_ represents the subset of the *i*-th sample with real labels, and $L_{i}^{*}$ represents the subset of the *i*-th sample with labels predicted and

$$\Delta (L_{i},L_{i}^{*})=\left\{ \begin{array}{c} 1,if\;L_{i}^{*}\;is\;identical\;to\;L_{i}\\ 0,other \end{array}\right.$$

statistical significance of differences between methods is quantified with the t-test.

### 2.5 Implementation details

Our prediction model was implemented using Tensorflow 1.12.0 and Keras 2.2.4. In the computer with an Intel(R) Xeon(R) CPU@2.20GHz and NVIDIA Titan XP GPU, it took about 2 hours to train the PrMFTP model, and PrMFTP took about 600 seconds for multi-functional therapeutic peptides prediction on test set. It is generally known that the performance of the DL model was affected by some hyperparameters, such as learning rate, number of hidden layers, and dropout regularization [45]. These hyperparameters were optimized by grid search on the training set with 5-fold cross-validation to achieve an optimal model as shown in S1 Table. In the DNN layer, CNN was constructed using the Conv1D function in Keras. To extract the features of peptide sequences with different lengths, three convolutional kernel sizes ks ∈ {2, 3, 8} were selected. Then, we trained our model with Adam optimizer, batch size = 64 and epochs = 60. To eliminate the effects induced by the random initialization of the DL framework, we repeated the training of all models ten times, and the average scores were obtained as the final predicted results for a test sample.

## 3. Results and discussion

### 3.1 Comparison of multi-label models using classical ML and DL methods

**Fig 2:**
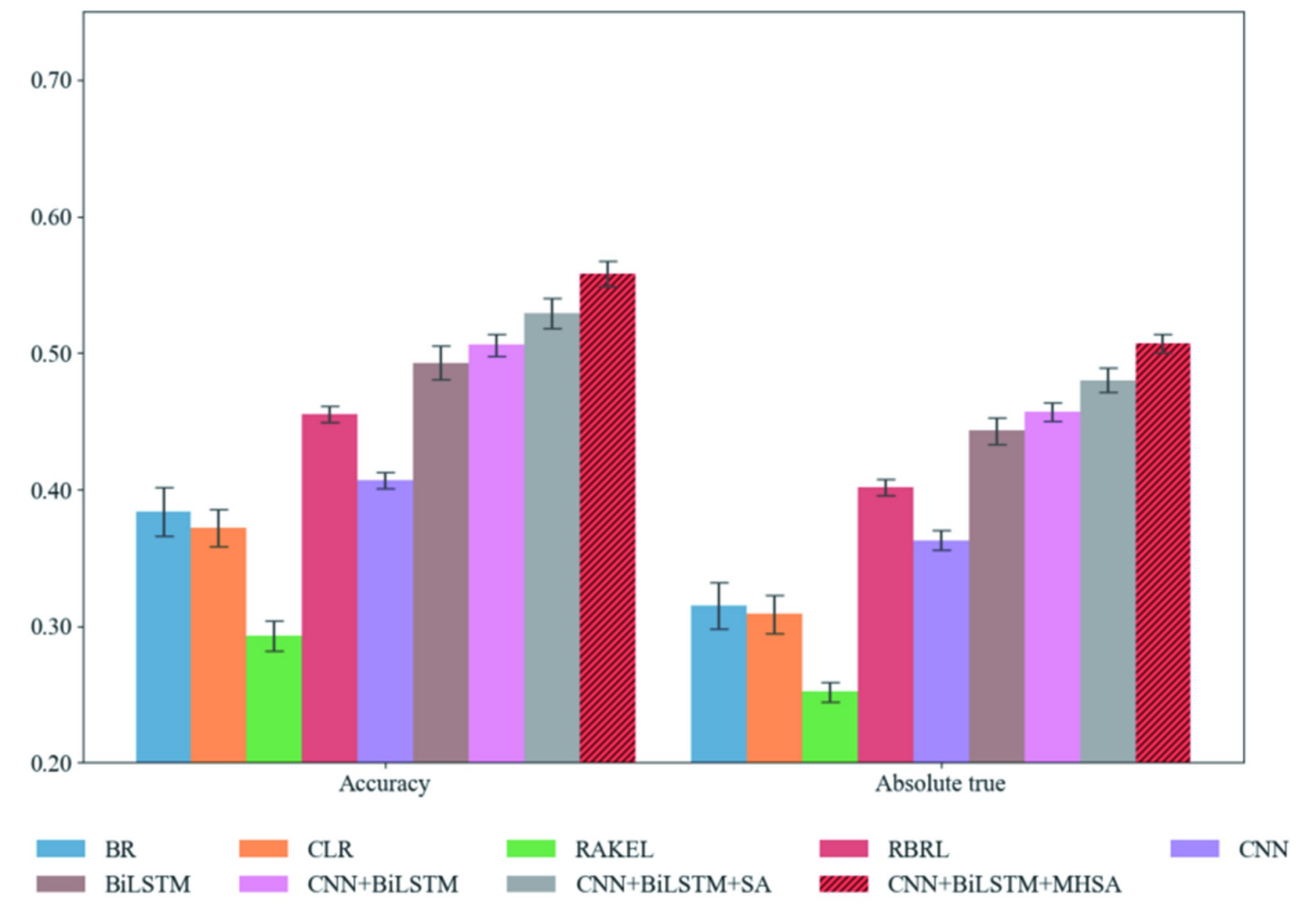
Accuracy and Absolute true of multi-label therapeutic peptides prediction models using different classical ML and DL algorithms on the training set with 5-fold cross-validation.

To achieve a high-effective model for multi-label therapeutic peptides prediction, we compared the performance of these models based on different classical ML methods (such as BR [28], CLR [29], random k-labelsets multi-label classification (RAKEL) [42] and RBRL [31]) and DL models (such as CNN, BiLSTM, CNN+BiLSTM, CNN+BiLSTM+SA). To ensure the fairness of model comparison, during the training processes for other ML and DL models, we employed two strategies: (1) the peptide sequences were uniformly encoded into digital vectors with a fixed dimension and served as input vectors for all models, and (2) we applied hyperparameter optimization for other ML and DL methods using a grid search method on the training with five-fold cross-validation. The classification performance of these models on the training set is presented in Fig 2 and S2 Table. As the more important metrics for multi-label classification evaluation, Accuracy and Absolute true were used to select the more perfect model. Fig 2 shows the average value of Accuracy and Absolute true on the training set, and we can see that CNN+BiLSTM+MHSA model has the best performance compared with other models. S2 Table shows that our model (CNN+BiLSTM+MHSA) is significantly on the training set with 5-fold cross-validation. CNN and BiLSTM in the DNN layer are used for local and global feature extraction, meanwhile, MHSA is used for further feature filtering. In addition, the performance of models based on DL is generally higher than that of models using classical ML. DL model automatically extracts the implicit feature information of peptide sequence to improve the performance of the model.

The performance of these models on the test set is shown in Table 2, which is similar with that on the training set. Compared with models based on CNN, BiLSTM and CNN+BiLSTM, our CNN+BiLSTM+MHSA model has 4.8% higher for Accuracy and 5.2% higher for Absolute true on the test set. Our model used the MHSA to optimize the feature matrix extracted from DNN layer. Compared with SA, the Accuracy and Absolute true of the model are improved by 3.8% and 3.7%, respectively. Therefore, we applied CNN+BiLSTM+MHSA model for multi-functional therapeutic peptides prediction.

**Table 2:** The performance of multi-label therapeutic peptides prediction models on the test set. The highest value is highlighted in bold.

Model	Precision ↑	Coverage ↑	Accuracy ↑	Absolute true ↑	Absolute false ↓
BR	0.427	0.437	0.394	0.325	0.050
CLR	0.418	0.428	0.387	0.320	0.047
RAKEL	0.349	0.317	0.307	0.265	0.052
RBRL	0.513	0.507	0.478	0.426	0.057
CNN	0.456	0.410	0.406	0.366	0.042
BiLSTM	0.572	0.532	0.522	0.472	0.037
CNN+BiLSTM	0.563	0.509	0.505	0.458	0.037
CNN+BiLSTM+SA	0.589	0.535	0.532	0.487	0.036
CNN+BiLSTM+MHSA (our model)	**0.626**	**0.574**	**0.570**	**0.524**	**0.034**

### 3.2 Comparison with classical algorithms for solving the problem of imbalanced data classification

**Fig 3:**
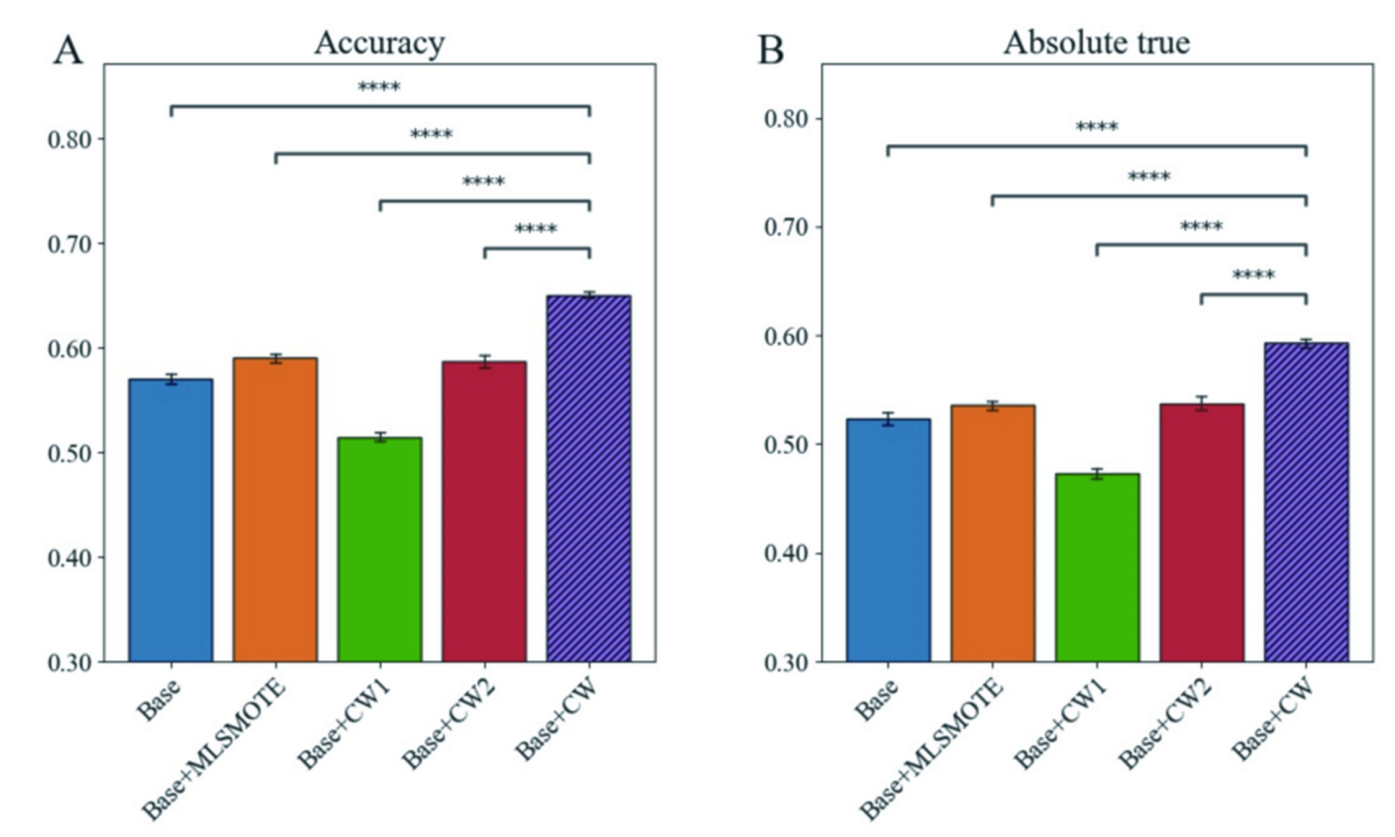
The performance comparison of the base (CNN+BiLSTM+MHSA) model with different algorithms for solving the problem of imbalanced data classification on the test set. A. Accuracy; B. Absolute true. Base+CW (our model) is significantly better at P-value < 0.001 (t-test).

Considering the high imbalanced level in the benchmark dataset, the undersampling method is easy to cause the loss of label information, especially the peptides with multiple labels. The oversampling method increases the size of peptide data with a small numbers, but it may affect other labels and lead to overfitting [46]. As a cost-sensitive approach, class weight optimization has been used to handle the imbalanced problem in the multi-label dataset [47]. In the previous studies, two methods [48] [46] have been proposed and applied to improve the multi-label classification performance (here we called these methods as CW1 and CW2, respectively). Considering the successes of CW1 and CW2, we employed these two class weight optimization methods in this study. In addition, we proposed CW as the third method. To estimate the improvement based on these three methods, we compared the values of Accuracy and Absolute true among the base model with different class weight optimization methods (CW1, CW2, and CW) on the same test subsets. We randomly extracted 80% of the test set results and repeated them five times to obtain five test subsets. The average values of Accuracy and Absolute true on the test set is shown in Fig 3, and the other metrics of the predicted results on the test set are in S3 Table. The results indicate that the model base+CW is superior to the other base models (base, base+CW1, base+CW2), and achieves the highest performance improvement.

The performance of these models is associated with class weight values, so we investigated the distribution of the class weight values of CW1, CW2 and CW methods for each label of therapeutic peptides. The class weights calculated by different methods are shown in S2 Fig. Comparing with the CW1 and CW2 methods, the CW method makes the value range of class weights and the difference among these class weights in relatively reasonable regions. Moreover, we can find the optimal depth learning parameters in the process of model training by increasing the weight of each class. Compared with CW2 and CW, CW1 set a higher weight for the classes with the smaller numbers. It will result in the decline of the overall performance of the model. By comparing the base+CW2 and base+CW models with the base model, it can be seen that the method of adding class weight can deal with the imbalance of multi-label data, and improve the performance. Finally, the base model combined with CW, named PrMFTP, was used for multi-functional therapeutic peptides prediction.

To further verify the superiority of CW method, MLSMOTE [49], a variation of SMOTE for multi-label sets, has been used to compare with our CW method. The results are shown in S3 Table, and it is found that CW method achieves better performance(Precision = 0.699, Coverage = 0.669, Accuracy = 0.651, Absolute true = 0.593 and Absolute false = 0.031)than MLSMOTE (Precision = 0.638, Coverage = 0.606, Accuracy = 0.591, Absolute true = 0.536 and Absolute false = 0.033) on the test set. As an oversampling method, MLSMOTE can increase the data size of minority labels, but may affect other labels and lead to overfitting. As a cost-sensitive method, CW considers higher costs for the misclassification of minority classes to handle the multi-label imbalanced data and improve the model performance.

### 3.3 Performance comparison of PrMFTP with the existing methods

At present, there are few methods to predict the MFTP, including TP-MV [50], MLBP. Although TP-MV is a therapeutic peptides prediction, PrMFTP cannot compare with this method. It is because that TP-MV used binary relevance to transform the multi-label task to more binary problems for specific functional peptides prediction. Our PrMFTP applied algorithm adaptation to construct a general model effectively and could be used for any functional peptide identification. Given abovementioned reason, we compared PrMFTP with MLBP, not TP-MV.

**Fig 4:**
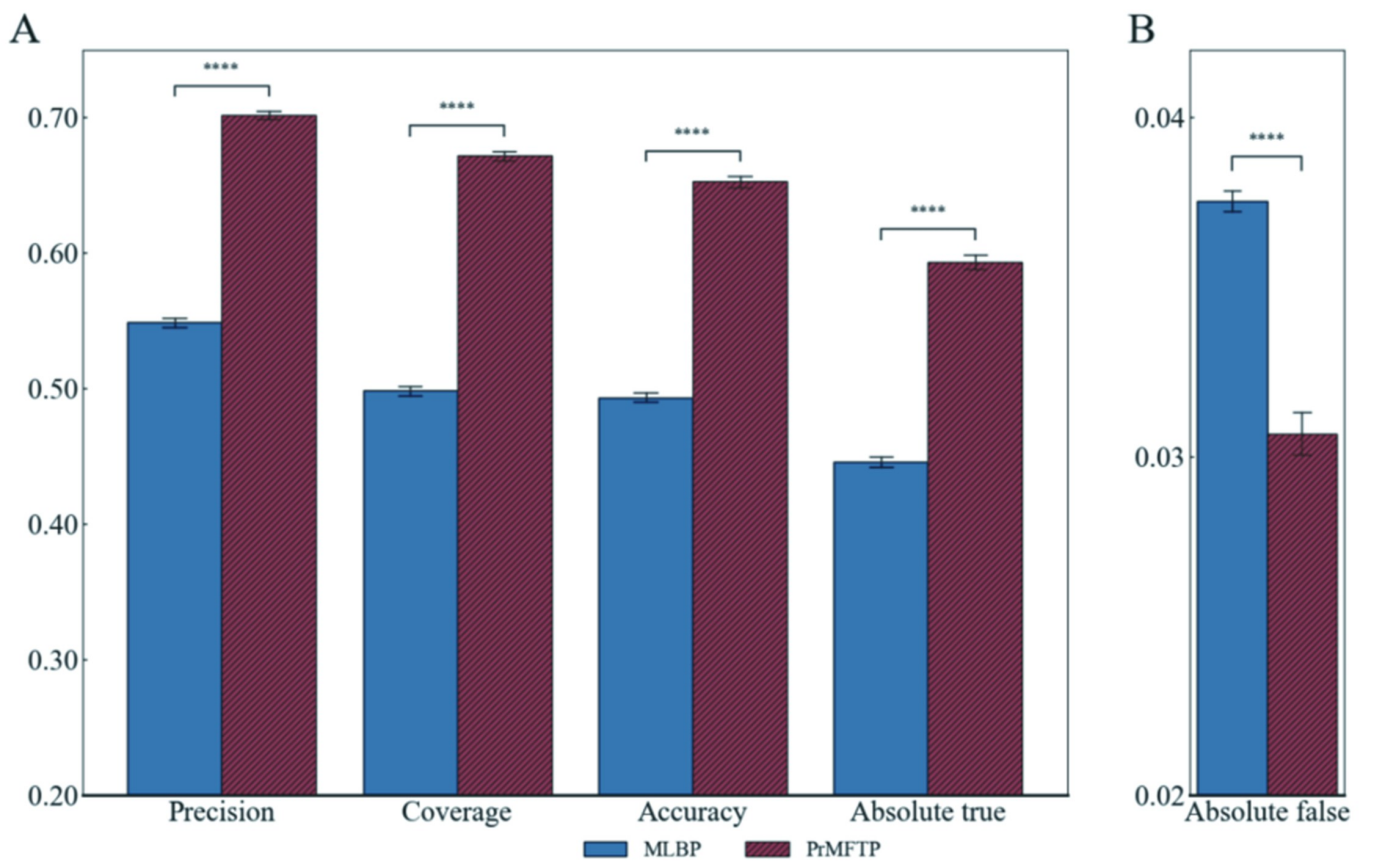
Performance comparison of MLBP and PrMFTP. A. Precision, Coverage, Accuracy and Absolute true; B. Absolute false. **** mean that PrMFTP is significantly better at P-value < 0.0001 (t-test).

MLBP based on multi-label DL method, MLBP was used to identify the multi-functional peptides of bioactive peptides, which can simultaneously predict multiple functional peptides including ACP, ADP, AHP, AIP, and AMP [4]. To further evaluate the performance of PrMFTP, we compared PrMFTP with MLBP. To ensure the fairness of the comparison, we retrained the model MLBP on our training set and compared the performance on the same test subsets. We randomly extracted 80% of the test set results and repeated them five times to obtain five test subsets. The average value of Precision, Coverage, Accuracy, Absolute true, and Absolute false for the five test subsets are shown in Fig 4. The result indicates that PrMFTP is superior to MLBP on all evaluation metrics. For example, Accuracy and Absolute true of PrMFTP were increased by 16.0% and 14.8%, respectively. It was noteworthy that MHSA in the PrMFTP model could further filter and optimize the features, and PrMFTP solved the problem of data imbalance through the optimization of class weight to improve the prediction performance of the model. To sum up, PrMFTP has a comparatively excellent performance.

### 3.4 Ablation study

According to the comparison of PrMFTP and MLBP (Fig 4), we discovered the importance of MHSA and class weight optimization on performance improvement. To further investigate the importance of multi-scale CNN, BiLSTM, MHSA, and CW in PrMFTP, we illustrated the role of these components through ablation experiments and compared the following variants of PrMFTP:

- w/o CNN is a variant that does not use multi-scale CNN.
- w/o BiLSTM is a variant that does not use BiLSTM.
- w/o MHSA is a variant that does not use MHSA.
- w/o CW is a variant that does not use CW.

Table 3 shows the performance of PrMFTP and their variants on the performance on the same test subsets. We randomly extracted 80% of the test set results and repeated them five times to obtain five test subsets. As seen, the removal of any module in PrMFTP would induce the performance decreases. This result illustrates that each module is crucial to PrMFTP’s performance. On the test set, the performance of the w/o BiLSTM model decreased most drastically, and the Accuracy and Absolute true decreased by 9.4% and 8.7%, respectively, followed by the w/o CNN model (the Accuracy and Absolute true decreased by 7.5% and 7.4%, respectively), the w/o CW model (the Accuracy and Absolute true decreased by 8.0% and 6.9%, respectively), and the w/o MHSA model (the Accuracy and Absolute true decreased by 3.0% and 3.5%, respectively). Comparing the results of the w/o CW, w/o BiLSTM, and PrMFTP models, the features extracted by the DL layer are conducive to improving the prediction performance of the model. Comparing the results of w/o CW and PrMFTP models, adding class weights is beneficial to improve the performance of the model. Removing MHSA leads to the performance degradation of the model, which shows that MHSA can optimize the extracted features and improve the performance of the model.

**Table 3:** The performance of PrMFTP and their variants on the test set. The highest value is highlighted in bold. w/o is abbreviation of without. The mean ± standard deviation on 5-fold cross-validation is shown for models. *, **, *** and **** mean that PrMFTP is significantly better at P-value < 0.05, P-value < 0.01, P-value < 0.001 and P-value < 0.0001 (t-test), respectively.

Model	Precision ↑	Coverage ↑	Accuracy ↑	Absolute true ↑	Absolute false ↓
PrMFTP	**0.699±0.004**	**0.669±0.004**	**0.651±0.004**	**0.593±0.004**	**0.031±0.001**
w/o CNN	0.618±0.006****	0.598±0.006****	0.576±0.006****	0.519±0.006****	0.033±0.001***
w/o BiLSTM	0.605±0.005****	0.573±0.005****	0.557±0.004****	0.504±0.004****	0.034±0.001****
w/o MHSA	0.660±0.002****	0.651±0.002****	0.621±0.003****	0.558±0.004****	0.032±0.001*
w/o CW	0.629±0.004****	0.574±0.005****	0.571±0.005****	0.524±0.007****	0.035±0.001****

### 3.5 The PrMFTP web server

To facilitate the pre-screening of therapeutic peptides by researchers, we established a user-friendly web server for the PrMFTP model (http://bioinfo.ahu.edu.cn/ PrMFTP). In this web server, the user can input the FASTA formatted peptide sequences into the main box or upload a FASTA file containing peptide sequences. Then, the user could achieve the prediction results with the mailbox or on the webserver. After that, the user clicks the submit button and start the prediction of these unknown peptides. It needs only a few minutes for the predicted results.

## Conclusion

In this study, to address the multi-functional prediction of therapeutic peptides, we propose a new prediction model based on MHSA and class weight optimization, called PrMFTP. Compared with the existing multi-label methods, PrMFTP achieves the highest prediction performance. The pivotal part of PrMFTP model includes the global and local information extraction of the sequence through multi-scale CNN and BiLSTM, and then the optimization of sequence features through MHSA. In addition, PrMFTP model effectively solves the problem of data imbalance by adding class weight and optimizing the value of class weight to a certain extent. In the future development of MFTP prediction, we will consider how to further solve the imbalance of data sets and improve the prediction performance of the model.

## Supporting information

S1 Fig Number distribution of therapeutic peptides. The upset plot shows the detailed number of therapeutic peptides in each group. In the upset plot, the ordinate represents the number of peptides, while the abscissa represents the components of each group. The pie charts exhibit the label distribution of the therapeutic peptides.
(PDF)

S2 Fig Class weights calculated by different methods. (PDF)

S1 Table Parameter details of PrMFTP model. (DOCX)

S2 Table The performance of different multi-label models for therapeutic peptides prediction on the training set with 5-fold cross-validation. The highest value is highlighted in bold. *, **, *** and **** mean that CNN+BiLSTM+MHSA (our model) is significantly better at P-value < 0.05, P-value < 0.01, P-value < 0.001 and P-value < 0.0001 (t-test), respectively.
(DOCX)

S3 Table The performance of the base (CNN+BiLSTM+MHSA) model with different calculation class weight methods and MLSMOTE on the test set. The highest value is highlighted in bold. On all performance metrics, Base+CW (our model) is significantly better compared with the other methods. The mean ± standard deviation on 5-fold cross-validation is shown for models. *, **, *** and **** mean that CNN+BiLSTM+MHSA (our model) is significantly better at P-value < 0.05, P-value < 0.01, P-value < 0.001 and P-value < 0.0001 (t-test), respectively.
(DOCX)

## References

[1] Markus Muttenthaler, King Glenn F, Adams David J, Alewood Paul F. Trends in peptide drug discovery. Nature Reviews Drug Discovery. 2021; 20:309–25. doi: 10.1038/s41573-020-00135-8 33536635

[2] Lei Wang, Nanxi Wang, Wenping Zhang, Xurui Cheng, Zhibin Yan, Gang Shao, et al. Therapeutic peptides: current applications and future directions. Signal Transduction and Targeted Therapy. 2022; 7:1–27. doi: 10.1038/s41392-022-00904-4 35165272 PMC8844085

[3] Haggag Yusuf A, Donia Ahmed A, Osman Mohamed A, El-Gizawy Sanaa A. Peptides as drug candidates: limitations and recent development perspectives. Biomedical Journal of Scientific & Technical Research. 2018; 1:3. 10.26717/bjstr.2018.08.001694

[4] Wending Tang, Ruyu Dai, Wenhui Yan, Wei Zhang, Yannan Bin, Enhua Xia, et al. Identifying multi-functional bioactive peptide functions using multi-label deep learning. Briefings in Bioinformatics. 2022; 23:bbab414. doi: 10.1093/bib/bbab414 34651655

[5] Deling Xu, Yanyan Wu, Zhixing Cheng, Jing Yang, Yanrui Ding. ACHP: a web server for predicting anti-cancer peptide and anti-hypertensive peptide. International Journal of Peptide Research and Therapeutics. 2021; 27:1933–44. 10.1007/s10989-021-10222-y

[6] Jinhao Zhang, Zehua Zhang, Lianrong Pu, Jijun Tang, Fei Guo. AIEpred: an ensemble predictive model of classifier chain to identify anti-inflammatory peptides. IEEE/ACM Transactions on Computational Biology and Bioinformatics. 2021; 18:1831–40. doi: 10.1109/TCBB.2020.2968419 31985437

[7] Ruyu Dai, Wei Zhang, Wending Tang, Evelien Wynendaele, Qizhi Zhu, Yannan Bin, et al. BBPpred: sequence-based prediction of blood-brain barrier peptides with feature representation learning and logistic regression. Journal of Chemical Information and Modeling. 2021; 61:525–34. doi: 10.1021/acs.jcim.0c01115 33426873

[8] Fallah Atanaki Fereshteh Behrouzi Saman, Shohreh Ariaeenejad, Amin Boroomand, Kaveh Kavousi. BIPEP: sequence-based prediction of biofilm inhibitory peptides using a combination of NMR and physicochemical descriptors. ACS Omega. 2020; 5:7290–7. doi: 10.1021/acsomega.9b04119 32280870 PMC7144140

[9] Yuxuan Pang, Zhuo Wang, Jhong Jhih-Hua Lee Tzong-Yi. Identifying anti-coronavirus peptides by incorporating different negative datasets and imbalanced learning strategies. Briefings in Bioinformatics. 2021; 22:1085–95. doi: 10.1093/bib/bbaa423 33497434 PMC7929366

[10] Muhammad Arif, Saeed Ahmad, Farman Ali, Ge Fang, Min Li, Dongjun Yu. TargetCPP: accurate prediction of cell-penetrating peptides from optimized multi-scale features using gradient boost decision tree. Journal of Computer-Aided Molecular Design. 2020; 34:841–56. doi: 10.1007/s10822-020-00307-z 32180124

[11] Watshara Shoombuatong, Nalini Schaduangrat, Reny Pratiwi, Chanin Nantasenamat. THPep: a machine learning-based approach for predicting tumor homing peptides. Computational Biology and Chemistry. 2019; 80:441–51. doi: 10.1016/j.compbiolchem.2019.05.008 31151025

[12] Piyush Agrawal, Dhruv Bhagat, Manish Mahalwal, Neelam Sharma, Raghava Gajendra PS. AntiCP 2.0: an updated model for predicting anticancer peptides. Briefings in Bioinformatics. 2021; 22:bbaa153. doi: 10.1093/bib/bbaa153 32770192

[13] Wei Zhang, Enhua Xia, Ruyu Dai, Wending Tang, Yannan Bin, Junfeng Xia. PredAPP: predicting anti-parasitic peptides with undersampling and ensemble approaches. Interdisciplinary Sciences: Computational Life Sciences. 2021:1–11. doi: 10.1007/s12539-021-00484-x 34608613

[14] Ke Yan, Hongwu Lv, Yichen Guo, Yongyong Chen, Hao Wu, Bin Liu. TPpred-ATMV: therapeutic peptide prediction by adaptive multi-view tensor learning model. Bioinformatics. 2022; 38:2712–8. doi: 10.1093/bioinformatics/btac200 35561206

[15] Li Hong-Liang Pang Yi-He, Bin Liu. BioSeq-BLM: a platform for analyzing DNA, RNA and protein sequences based on biological language models. Nucleic Acids Research. 2021; 49:e129. doi: 10.1093/nar/gkab829 34581805 PMC8682797

[16] Youngmahn Han, Dongsup Kim. Deep convolutional neural networks for pan-specific peptide-MHC class I binding prediction. BMC Bioinformatics. 2017; 18:1–9. doi: 10.1186/s12859-017-1997-x 29281985 PMC5745637

[17] Matt Spencer, Jesse Eickholt, Jianlin Cheng. A deep learning network approach to ab initio protein secondary structure prediction. IEEE/ACM Transactions on Computational Biology and Bioinformatics. 2014; 12:103–12. doi: 10.1109/TCBB.2014.2343960 25750595 PMC4348072

[18] Sønderby Kaae Søren, Sønderby Casper Kaae Nielsen Henrik, Ole Winther, editors. Convolutional LSTM networks for subcellular localization of proteins. International Conference on Algorithms for Computational Biology; 2015. 10.1007/978-3-319-21233-3_6

[19] Chu Y, Kaushik A. C, Wang X, Wang W, Zhang Y, Shan X, et al. DTI-CDF: a cascade deep forest model towards the prediction of drug-target interactions based on hybrid features. Briefings in Bioinformatics. 2021; 22:451–62. doi: 10.1093/bib/bbz152 31885041

[20] Deng Y, Xu X, Qiu Y, Xia J, Zhang W, Liu S. A multimodal deep learning framework for predicting drug-drug interaction events. Bioinformatics. 2020; 36:4316–22. doi: 10.1093/bioinformatics/btaa501 32407508

[21] Huang F, Yue X, Xiong Z, Yu Z, Liu S, Zhang W. Tensor decomposition with relational constraints for predicting multiple types of microRNA-disease associations. Briefings in Bioinformatics. 2021; 22:bbaa140. doi: 10.1093/bib/bbaa140 32725161

[22] Lin Tzu-Tang Yang Li-Yen, Lu I-Hsuan Cheng Wen-Chih, Hsu Zhe-Ren Chen Shu-Hwa, et al. AI4AMP: an antimicrobial peptide predictor using physicochemical property-based encoding method and deep Learning. Msystems. 2021; 6:e00299–21. doi: 10.1128/mSystems.00299-21 34783578 PMC8594441

[23] Ritesh Sharma, Sameer Shrivastava, Kumar Singh Sanjay Kumar Abhinav, Sonal Saxena, Kumar Singh Raj. Deep-ABPpred: identifying antibacterial peptides in protein sequences using bidirectional LSTM with word2vec. Briefings in Bioinformatics. 2021; 22:bbab065. doi: 10.1093/bib/bbab065 33784381

[24] Chun Fang, Yoshitaka Moriwaki, Caihong Li, Kentaro Shimizu. Prediction of antifungal peptides by deep learning with character embedding. IPSJ Transactions on Bioinformatics. 2019; 12:21–9. 10.2197/ipsjtbio.12.21

[25] Jiawei Li, Yuqian Pu, Jijun Tang, Quan Zou, Fei Guo. DeepAVP: a dual-channel deep neural network for identifying variable-length antiviral peptides. IEEE Journal of Biomedical and Health Informatics. 2020; 24:3012–9. doi: 10.1109/JBHI.2020.2977091 32142462

[26] Shumin Li, Junjie Chen, Bin Liu. Protein remote homology detection based on bidirectional long short-term memory. BMC Bioinformatics. 2017; 18 doi: 10.1186/s12859-017-1842-2 29017445 PMC5634958

[27] Minling Zhang, Zhihua Zhou. A review on multi-label learning algorithms. IEEE Transactions on Knowledge and Data Engineering. 2013; 26:1819–37. 10.1109/tkde.2013.39

[28] Boutell Matthew R, Luo Jiebo, Shen Xipeng, Brown Christopher M. Learning multi-label scene classification. Pattern Recognition. 2004; 37:1757–71. 10.1016/j.patcog.2004.03.009

[29] Johannes Fürnkranz, Eyke Hüllermeier, Loza Mencía Eneldo Brinker Klaus. Multilabel classification via calibrated label ranking. Machine Learning. 2008; 73:133–53. 10.1007/s10994-008-5064-8

[30] Grigorios Tsoumakas, Ioannis Vlahavas. Random k-labelsets: an ensemble method for multilabel classification. European Conference on Machine Learning. 2007:406–17. 10.1007/978-3-540-74958-5_38

[31] Wu Guoqiang, Zheng Ruobing, Tian Yingjie, Liu Dalian. Joint ranking SVM and binary relevance with robust low-rank learning for multi-label classification. Neural Networks. 2020; 122:24–39. doi: 10.1016/j.neunet.2019.10.002 31675625

[32] Zhouhan Lin., Feng Minwei., Santos Cicero Nogueira dos., Yu., Xiang Bing., Zhou Bowen., et al. A structured self-attentive sentence embedding. ArXiv Preprint ArXiv:170303130. 2017; 10.48550/arXiv.1703.03130. Focus to learn more

[33] Zhang Yu P, Zou Quan. PPTPP: a novel therapeutic peptide prediction method using physicochemical property encoding and adaptive feature representation learning. Bioinformatics. 2020; 36:3982–7. doi: 10.1093/bioinformatics/btaa275 32348463

[34] Xuan Xiao, Yutao Shao, Xiang Cheng, Biljana Stamatovic. iAMP-CA2L: a new CNN-BiLSTM-SVM classifier based on cellular automata image for identifying antimicrobial peptides and their functional types. Briefings in Bioinformatics. 2021; 22:bbab209. doi: 10.1093/bib/bbab209 34086856

[35] Susanta Roy, Robindra Teron. BioDADPep: a bioinformatics database for anti diabetic peptides. Bioinformation. 2019; 15:780. doi: 10.6026/97320630015780 31902976 PMC6936660

[36] Mst Khatun, Md Hasan, Watshara Shoombuatong, Hiroyuki Kurata. ProIn-Fuse: improved and robust prediction of proinflammatory peptides by fusing of multiple feature representations. Journal of Computer-Aided Molecular Design. 2020; 34:1229–36. doi: 10.1007/s10822-020-00343-9 32964284

[37] Pankhuri Jain, Tiwari Anoop Kumar Som Tanmoy. Enhanced prediction of anti-tubercular peptides from sequence information using divergence measure-based intuitionistic fuzzy-rough feature selection. Soft Computing. 2020; 25:3065–86. 10.1007/s00500-020-05363-z

[38] Phasit Charoenkwan, Sakawrat Kanthawong, Chanin Nantasenamat, Hasan Md Mehedi Shoombuatong Watshara. IDPPIV-SCM: a sequence-based predictor for identifying and analyzing dipeptidyl peptidase IV (DPP-IV) inhibitory peptides using a scoring card method. Journal of Proteome Research. 2020; 19:4125–36. doi: 10.1021/acs.jproteome.0c00590 32897718

[39] Hyun Kim, Jang Ju Hye Kim Sun Chang, Cho Ju Hyun. De novo generation of short antimicrobial peptides with enhanced stability and cell specificity. Journal of Antimicrobial Chemotherapy. 2014; 69:121–32. doi: 10.1093/jac/dkt322 23946320

[40] Eugen Vušak, Vjeko Kužina, Alan Jović, editors. A survey of word embedding algorithms for textual data information extraction. 2021 44th International Convention on Information, Communication and Electronic Technology (MIPRO): IEEE. 10.23919/mipro52101.2021.9597076

[41] Aslan Muhammet Fatih Unlersen Muhammed Fahri, Kadir Sabanci, Akif Durdu. CNN-based transfer learning-BiLSTM network: a novel approach for COVID-19 infection detection. Applied Soft Computing. 2021; 98:106912. doi: 10.1016/j.asoc.2020.106912 33230395 PMC7673219

[42] Jianpeng Zhou, Lei Chen, Zihan Guo. iATC-NRAKEL: an efficient multi-label classifier for recognizing anatomical therapeutic chemical classes of drugs. Bioinformatics. 2020; 36:1391–6. doi: 10.1093/bioinformatics/btz757 31593226

[43] Sadaf Gull, Nauman Shamim, Fayyaz Minhas. AMAP: hierarchical multi-label prediction of biologically active and antimicrobial peptides. Computers in Biology and Medicine. 2019; 107:172–81. doi: 10.1016/j.compbiomed.2019.02.018 30831306

[44] Xuan Xiao, Pu Wang, Weizhong Lin, Jianhua Jia, Kuochen Chou. iAMP-2L: a two-level multi-label classifier for identifying antimicrobial peptides and their functional types. Analytical Biochemistry. 2013; 436:168–77. doi: 10.1016/j.ab.2013.01.019 23395824

[45] Alexios Koutsoukas, Monaghan Keith J Li Xiaoli, Jun Huan. Deep-learning: investigating deep neural networks hyper-parameters and comparison of performance to shallow methods for modeling bioactivity data. Journal of Cheminformatics. 2017; 9:1–13. doi: 10.1186/s13321-017-0226-y 29086090 PMC5489441

[46] Konstantin Sozykin, Stanislav Protasov, Adil Khan, Rasheed Hussain, Jooyoung Lee. Multi-label class-imbalanced action recognition in hockey videos via 3D convolutional neural networks. 2018 19th IEEE/ACIS International Conference on Software Engineering, Artificial Intelligence, Networking and Parallel/Distributed Computing (SNPD). 2018:146–51. 10.1109/snpd.2018.8441034

[47] Tarekegn Adane Nega, Mario Giacobini, Michalak Krzysztof. A review of methods for imbalanced multi-label classification. Pattern Recognition. 2021; 118:107965. 10.1016/j.patcog.2021.107965

[48] Duolin Wang, Zhaoyue Zhang, Yuexu Jiang, Ziting Mao, Dong Wang, Hao Lin, et al. DM3Loc: multi-label mRNA subcellular localization prediction and analysis based on multi-head self-attention mechanism. Nucleic Acids Research. 2021; 49:e46. doi: 10.1093/nar/gkab016 33503258 PMC8096227

[49] Francisco Charte, Rivera Antonio J, del Jesus María J, Herrera Francisco. MLSMOTE: Approaching imbalanced multilabel learning through synthetic instance generation. Knowledge-Based Systems. 2015; 89:385–97. 10.1016/j.knosys.2015.07.019

[50] Ke Yan, Hongwu Lv, Jie Wen, Yichen Guo, Bin Liu. TP-MV: Therapeutic Peptides Prediction by Multi-view Learning. Current Bioinformatics. 2022; 17:174–83. 10.2174/1574893617666211220153429

