# Supplementary figures and images for: PrMFTP: Multi-functional therapeutic peptides prediction based on multi-head self-attention mechanism and class weight optimization
Source: PLoS Comput Biol. 2022 Sep 12;18(9):e1010511. doi: 10.1371/journal.pcbi.1010511 (PMC9499272; doi:10.1371/journal.pcbi.1010511)

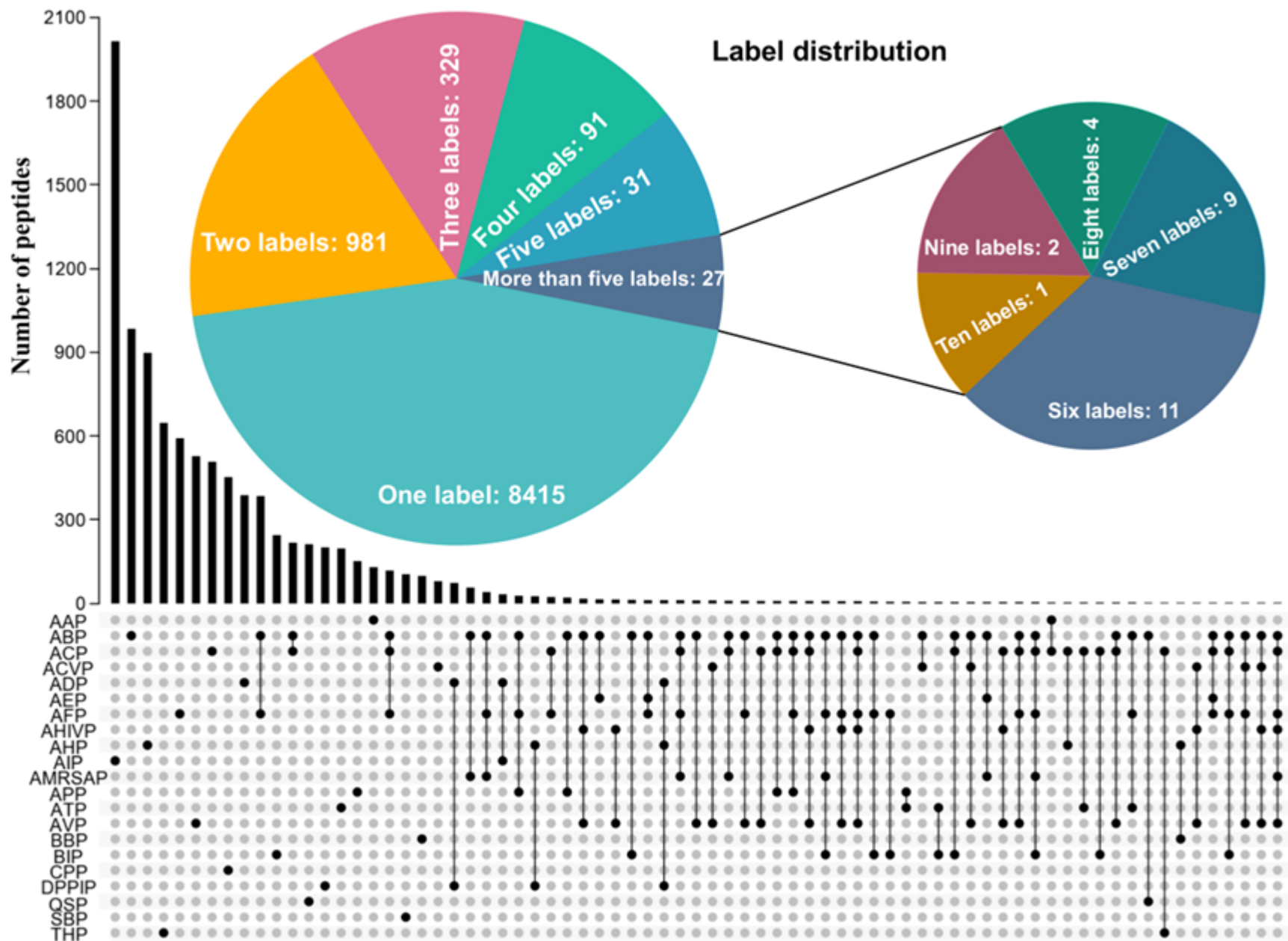

Supplement: S1 Fig — The upset plot shows the detailed number of therapeutic peptides in each group. In the upset plot, the ordinate represents the number of peptides, while the abscissa represents the components of each group. The pie charts exhibit the label distribution of the therapeutic peptides. (PDF) [file pcbi.1010511.s001.pdf]

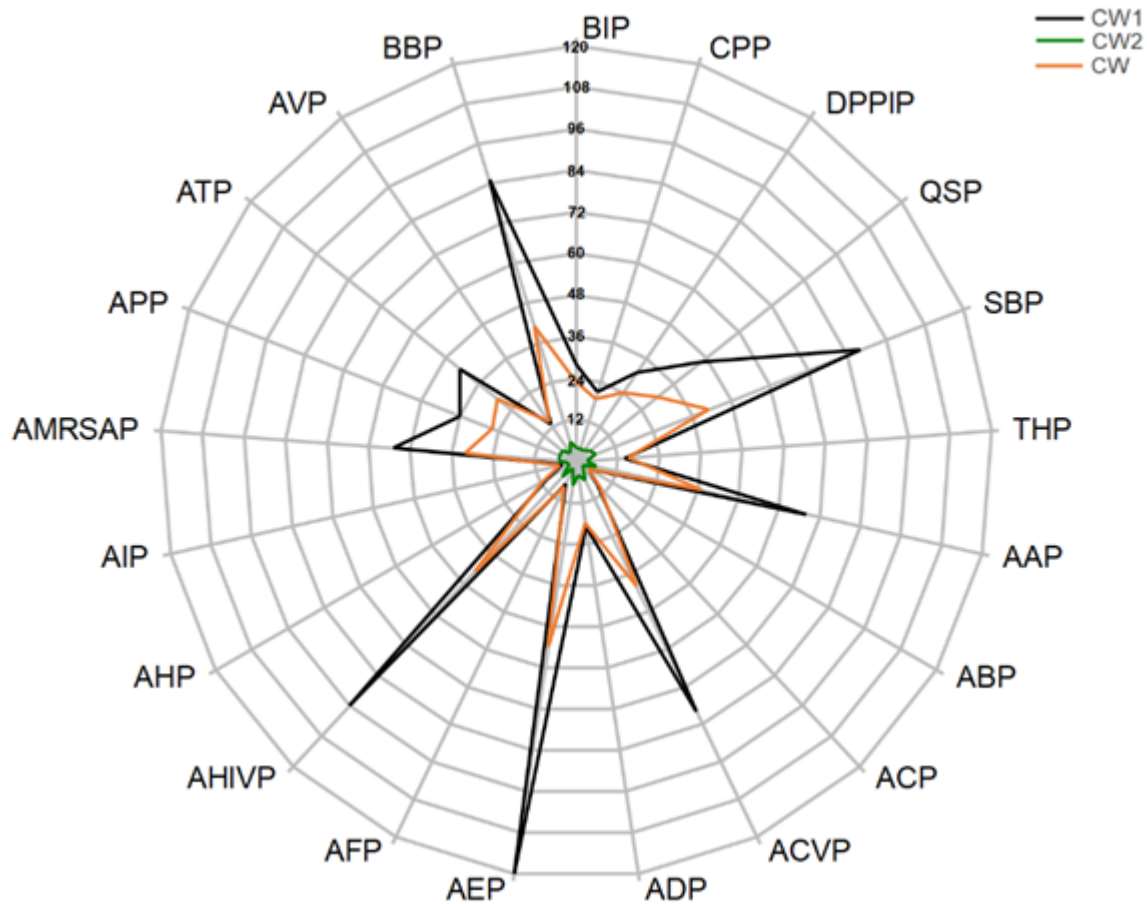

Supplement: S2 Fig — (PDF) [file pcbi.1010511.s002.pdf]
