# Supplementary material for: PrMFTP: Multi-functional therapeutic peptides prediction based on multi-head self-attention mechanism and class weight optimization
Source: PLoS Comput Biol. 2022 Sep 12;18(9):e1010511. doi: 10.1371/journal.pcbi.1010511 (PMC9499272; doi:10.1371/journal.pcbi.1010511)
Supplement: S1 Table — (DOCX) [file pcbi.1010511.s003.docx]

**S1 Table. Parameter details of PrMFTP model.**

| Module | Parameter | Scope | Optimal |
| --- | --- | --- | --- |
| Embedding module | Embedding dimension | (64, 128, 192) | 128 |
| Multi-scale CNN | Pooling size | (3, 5) | 3 |
| BiLSTM module | LSTM unit | (50, 100, 150) | 100 |
| MHSA module | Attention head size | (5, 8) | 5 |
|  | Attention dimension | (80, 100) | 80 |
| Classification module | Fully connected dimension | (64, 128) | 128 |
| Training module | Learning rate | (0.01, 0.001) | 0.001 |
