# Supplementary material for: PrMFTP: Multi-functional therapeutic peptides prediction based on multi-head self-attention mechanism and class weight optimization
Source: PLoS Comput Biol. 2022 Sep 12;18(9):e1010511. doi: 10.1371/journal.pcbi.1010511 (PMC9499272; doi:10.1371/journal.pcbi.1010511)
Supplement: S2 Table — The highest value is highlighted in bold. *, **, *** and **** mean that CNN+BiLSTM+MHSA (our model) is significantly better at P-value < 0.05, P-value < 0.01, P-value < 0.001 and P-value < 0.0001 (t-test), respectively. (DOCX) [file pcbi.1010511.s004.docx]

**S2 Table. The performance of different multi-label models for therapeutic peptides prediction on the training set with 5-fold cross-validation.** The highest value is highlighted in bold. The mean ± standard deviation on 5-fold cross-validation is shown for models. *, **, *** and **** mean that CNN+BiLSTM+MHSA (our model) is significantly better at P-value < 0.05, P-value < 0.01, P-value < 0.001 and P-value < 0.0001 (t-test), respectively.

| **Model** | **Precision ↑** | **Coverage ↑** | **Accuracy ↑** | **Absolute true ↑** | **Absolute false ↓** |
| --- | --- | --- | --- | --- | --- |
| BR | 0.419±0.018^****^ | 0.426±0.018^****^ | 0.384±0.018^****^ | 0.315±0.017^****^ | 0.052±0.001^****^ |
| CLR | 0.405±0.014^****^ | 0.410±0.015^****^ | 0.372±0.014^****^ | 0.309±0.014^****^ | 0.049±0.001^****^ |
| RAKEL | 0.336±0.013^****^ | 0.301±0.012^****^ | 0.293±0.011^****^ | 0.252±0.007^****^ | 0.052±0.001^****^ |
| RBRL | 0.492±0.006^****^ | 0.481±0.006^****^ | 0.455±0.006^****^ | 0.402±0.006^****^ | 0.060±0.001^****^ |
| CNN | 0.459±0.008^****^ | 0.411±0.007^****^ | 0.407±0.006^****^ | 0.363±0.007^****^ | 0.043±0.001^****^ |
| BiLSTM | 0.545±0.015^****^ | 0.502±0.012^****^ | 0.493±0.012^****^ | 0.443±0.010^****^ | 0.039±0.001^**^ |
| CNN+BiLSTM | 0.564±0.013^***^ | 0.511±0.007^****^ | 0.506±0.008^****^ | 0.457±0.007^****^ | 0.038±0.001^**^ |
| CNN+BiLSTM+SA | 0.585±0.016^**^ | 0.535±0.011^**^ | 0.529±0.011^**^ | 0.480±0.009^***^ | 0.037±0.001^*^ |
| CNN+BiLSTM+ MHSA (our model) | **0.614±0.012** | **0.565±0.008** | **0.558±0.009** | **0.507±0.007** | **0.035±0.001** |
