# Supplementary material for: PrMFTP: Multi-functional therapeutic peptides prediction based on multi-head self-attention mechanism and class weight optimization
Source: PLoS Comput Biol. 2022 Sep 12;18(9):e1010511. doi: 10.1371/journal.pcbi.1010511 (PMC9499272; doi:10.1371/journal.pcbi.1010511)
Supplement: S3 Table — The highest value is highlighted in bold. On all performance metrics, Base+CW (our model) is significantly better compared with the other methods. The mean ± standard deviation on 5-fold cross-validation is shown for models. *, **, *** and **** mean that CNN+BiLSTM+MHSA (our model) is significantly better at P-value < 0.05, P-value < 0.01, P-value < 0.001 and P-value < 0.0001 (t-test), respectively. (DOCX) [file pcbi.1010511.s005.docx]

**S3 Table. The performance of the base (CNN+BiLSTM+MHSA) model with different algorithms for solving the problem of imbalanced data classification on the test set.** The highest value is highlighted in bold. On all performance metrics, Base+CW (our model) is significantly better compared with the other methods. The mean ± standard deviation on 5-fold cross-validation is shown for models. *, **, *** and **** mean that CNN+BiLSTM+MHSA (our model) is significantly better at P-value < 0.05, P-value < 0.01, P-value < 0.001 and P-value < 0.0001 (t-test), respectively.

| **Model** | **Precision ↑** | **Coverage ↑** | **Accuracy ↑** | **Absolute true ↑** | **Absolute false ↓** |
| --- | --- | --- | --- | --- | --- |
| Base | 0.629±0.004^****^ | 0.574±0.005^****^ | 0.571±0.005^****^ | 0.524±0.007^****^ | 0.035±0.001^****^ |
| Base+MLSMOTE | 0.638±0.006^****^ | 0.606±0.005^****^ | 0.591±0.005^****^ | 0.536±0.004^****^ | 0.033±0.001^***^ |
| Base+CW1 | 0.559±0.006^****^ | 0.522±0.005^****^ | 0.515±0.005^****^ | 0.473±0.005^****^ | 0.036±0.001^****^ |
| Base+CW2 | 0.640±0.007^****^ | 0.594±0.007^****^ | 0.587±0.007^****^ | 0.538±0.007^****^ | 0.033±0.001^**^ |
| Base+CW | **0.699±0.004** | **0.669±0.004** | **0.651±0.004** | **0.593±0.004** | **0.031±0.001** |
